# Activation of TRESK channels by the inflammatory mediator lysophosphatidic acid balances nociceptive signalling

**DOI:** 10.1038/srep12548

**Published:** 2015-07-30

**Authors:** Sina Kollert, Benjamin Dombert, Frank Döring, Erhard Wischmeyer

**Affiliations:** 1 Institute of Physiology, AG Molecular Electrophysiology, University of Würzburg, 97070 Würzburg Germany; 2 Institute for Clinical Neurobiology, University Hospital Würzburg, 97078 Würzburg, Germany

## Abstract

In dorsal root ganglia (DRG) neurons TRESK channels constitute a major current component of the standing outward current IK_SO_. A prominent physiological role of TRESK has been attributed to pain sensation. During inflammation mediators of pain e.g. lysophosphatidic acid (LPA) are released and modulate nociception. We demonstrate co-expression of TRESK and LPA receptors in DRG neurons. Heterologous expression of TRESK and LPA receptors in *Xenopus* oocytes revealed augmentation of basal K^+^ currents upon LPA application. In DRG neurons nociception can result from TRPV1 activation by capsaicin or LPA. Upon co-expression in *Xenopus* oocytes LPA simultaneously increased both depolarising TRPV1 and hyperpolarising TRESK currents. Patch-clamp recordings in cultured DRG neurons from TRESK[wt] mice displayed increased IK_SO_ after application of LPA whereas under these conditions IK_SO_ in neurons from TRESK[ko] mice remained unaltered. Under current-clamp conditions LPA application differentially modulated excitability in these genotypes upon depolarising pulses. Spike frequency was attenuated in TRESK[wt] neurons and, in contrast, augmented in TRESK[ko] neurons. Accordingly, excitation of nociceptive neurons by LPA is balanced by co-activation of TRESK channels. Hence excitation of sensory neurons is strongly controlled by the activity of TRESK channels, which therefore are good candidates for the treatment of pain disorders.

The family of tandem-pore potassium (K2P) channels comprises 15 members that constitute background or leak currents and control cellular excitability. They are widely expressed in the nervous system and their activity is regulated by a plethora of extracellular and intracellular physiological messengers^1^. In addition, K2P channels are regulated by neurotransmitters and G-protein coupled receptors^2^. Rat brainstem motoneurons, for example, have been shown to be regulated by the neurotransmitter serotonin that inhibits the K2P channel TASK^3^ probably via the α-subunit of Gαq-coupled receptors^4^. Other studies have shown that diacylglycerol mediates the activity of TASK channels in transfected cell lines^5^ as well as in native neurons^6^.

In dorsal root ganglia (DRGs) and trigeminal peripheral neurons the K2P channels TREK-2 and TRESK (TWIK-related spinal cord potassium) are the major current components of the standing outward current IK_SO_^7^,^8^. A main physiological function of TRESK has been attributed to the modulation of nociception. Down-regulation of TRESK expression by siRNA increased the sensitivity to painful stimuli^9^. In line with these findings overexpression of TRESK in DRG neurons attenuates nerve injury-induced mechanical allodynia^10^. A frameshift mutation in the KCNK18 gene coding for TRESK has recently been shown to be involved in the development of a certain form of migraine with aura^11^. The truncated channel protein leads to a complete loss of TRESK function and, moreover, exerts a dominant negative effect on wildtype channels^12^. A key feature of TRESK channels is their activation by Gαq-coupled receptors. M1 cholinergic receptors potentiate TRESK currents up to 5-fold through an intracellular pathway including phospholipase C, calcium and calcineurin^13^.

Important physiological mediators of peripheral nociception are substances released during inflammation after tissue injury. These factors represent a wide array of signalling molecules, such as neurotransmitters (e.g. serotonin, histamine), peptides (e.g bradykinin), lipids, neurotrophins, cytokines and chemokines^14^. Only recently lysophosphatidic acid was found to play a major role in inflammatory disorders with direct accumulation at sites of inflammation^15^. Each of these factors sensitise or excite nociceptors by interacting with cell surface receptors expressed in these neurons^16^.

Lysophosphatidic acid (LPA) is a small, ubiquitous lysophospholipid that is released upon tissue injury^17^ and acts as an extracellular molecule by binding to and activating at least five known G protein-coupled receptors, LPA_1_-LPA_5_^18^. In the nervous system LPA signalling influences cortical development, survival, migration and proliferation of cells as well as neurological disorders such as schizophrenia and neuropathic pain^19^. However, its function on the molecular level is still poorly understood. During tissue injury LPA is released from activated platelets or microglia, thereby altering the activity of ion channels that regulate the excitability of neurons. In heterologous systems TREK-1, another prominent member of the K2P channel family, was shown to be down-modulated by LPA^20^. In primary nociceptors TRPV1 (transient receptor potential vanilloid receptor type 1) currents appear to be directly activated by this lipid^21^. Both channels account for significant current components in DRG neurons^5^,^7^,^22^.

In the present study we use heterologous gene expression to show that LPA strongly activates TRESK channels by its Gαq-coupled receptors. In primary DRG neurons the excitatory effect of LPA was shown to be balanced by co-activation of TRESK channels as revealed from differences in wildtype and functional TRESK knockout mice.

## Results

### Detection of TRESK channel protein and LPA receptor transcripts in DRG

#### TRESK antibody

**Figure 1:**
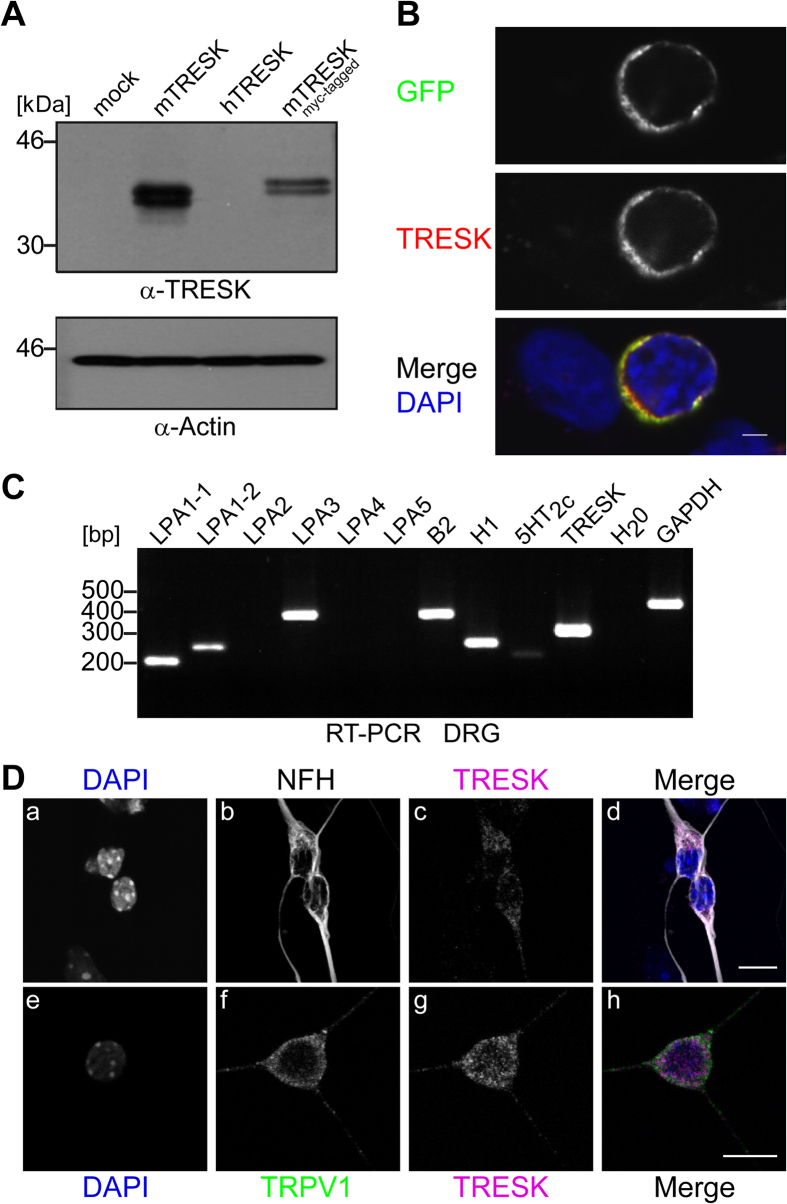
Expression profile of LPA receptors and immunodetection of TRESK channel in DRG neurons. (**A**) Whole cell extracts of HEK-293 cells transfected with pcDNA3 (mock), mTRESK, hTRESK and myc-mTRESK plasmid DNA (as indicated) were immunoblotted and analysed with polyclonal TRESK antibody (upper panel) or anti-Actin antibody as loading control (lower panel). Extracts of cells transfected with mTRESK or myc-mTRESK displayed double bands with the expected molecular weight indicating high specificity for mouse TRESK of the novel antibody. (**B**) HEK-293 cells were transfected with mTRESK-eGFP fusion construct and analysed by confocal microscopy. Only transfected cells (GFP in upper panel) were found to have membrane reactivity with TRESK antibodies (middle panel). Lower panel, merge displayed co-reactivity of the antibody with GFP signal. DAPI staining in blue revealed also non-transfected cells. Scale bar 2 μm. (**C**) Expression of mTRESK, and a selection of Gq-coupled receptors (as indicated) was monitored in adult DRGs by RT-PCR. Primers for GAPDH were used as a positive control and H_2_O was applied as a negative control in reverse transcription. (**D**) Immunocytochemistry of cultured DRG neurons from E13.5 mouse embryos probed with antibodies against TRESK (c, g), NFH (b), TRPV1 (f) and DAPI (a, e). Images a-c and e-g are merged in panel d and h, respectively. Merge in d documented that only NFH-stained neurons (grey) displayed TRESK reactivity (magenta); DAPI-stained, non-neuronal cells (a, upper and lower edge) displayed hardly any TRESK signal (c). Neuronal co-expression of TRESK (magenta) and TRPV1 (green) was documented in merge of panel h (out of 29 TRESK-positive cells 18 were also positive for TRPV1). Fused z-stacks of confocal images are shown. Scale bar 10 μm.

To detect TRESK channel protein in native cells we tested commercially available peptide antibodies (Alomone Labs Ltd., Santa Cruz Biotechnology, Abcam) for TRESK specificity. In our hands none of those was able to detect TRESK-specific signals by comparison of human embryonal kidney HEK-293 cells transfected with the channel or mock-transfected cells (data not shown). Thus we developed a polyclonal rabbit antibody directed against a polypeptide of 68 amino acids (Arg197-Ser264) within the intracellular loop between transmembrane segments M2 and M3 of mouse TRESK (mTRESK) subunit. Western blot analysis of whole cell protein extracts from mTRESK-transfected HEK-293 revealed a double band at the expected molecular weight of approx. 44 kD (compare 23). The same pattern at slightly higher molecular weight was found in extracts of HEK-293 cells transfected with myc-tagged mTRESK cDNA. However, recombinant human TRESK expressed in HEK-293 cells was not detected by the antibody and also no signals were found in control extracts of mock-transfected cells indicating the specificity of the antibody against mouse TRESK channels (Fig. 1A). Similarly, by immunocytochemistry antibody labelling was found only in HEK-293 cells transfected with mTRESK-GFP. Membrane staining of TRESK antibody and GFP display identical patterns as the fluorescent protein was fused to the C-terminus of the channel (Fig. 1B).

#### TRESK and LPA receptors in DRGs

TRESK channels are involved in nociceptive signalling, which is modulated under inflammatory conditions^24^,^25^. Pro-inflammatory mediators e.g. bradykinin or serotonin, as well as LPA are released during inflammation and thus the question arises if the corresponding receptors are co-expressed with TRESK channels in DRG neurons. Using RT-PCR with gene-specific primers, transcripts of H_1_, B_2_ and 5HT_2c_ receptors were detected in dorsal root ganglia from adult mouse. In addition a distinct set of LPA receptors, namely LPA_1-1_, LPA_1-2_ and LPA_3_, were found to be co-expressed with TRESK channels in this tissue (Fig. 1C) without resolution of different cell populations.

In preparations of primary cells from dissected embryonic DRGs, sensory neurons, glial cells and fibroblasts were isolated and grown together. To distinguish neurons from other cell populations, these cultures were immunostained with antibodies against neurofilament-H (NFH). Co-labelling with TRESK antibodies revealed specific dot-like signals in almost every NFH-positive cells, documenting a distinct expression of the channel, apparently with a moderate level of protein (Fig. 1D, panel a-d). TRESK immunreactivity was detected not only in neuronal cell bodies but also in the corresponding neurites (Fig. 1D, panel c and g), supporting the idea of its sensory function in the periphery. To verify if TRESK may in principle attenuate depolarisation by excitatory channels during nociception we conducted another double staining with TRPV1 and TRESK antibodies. Confocal imaging detected co-distribution of both channels in the soma as well as in neurites of the same neuron (Fig. 1D, panel e-h).

### Augmentation of TRESK currents by LPA receptor activation

Expression profiles of TRESK channels^24^ indicate their contribution to signal transduction during inflammatory processes. Thus we tested TRESK regulation by Gαq-coupled receptors mediated by several substances released upon inflammation. Initially, receptors of well-known mediators like bradykinin, serotonin or histamine were co-expressed with TRESK channels in *Xenopus* oocytes.

**Figure 2:**
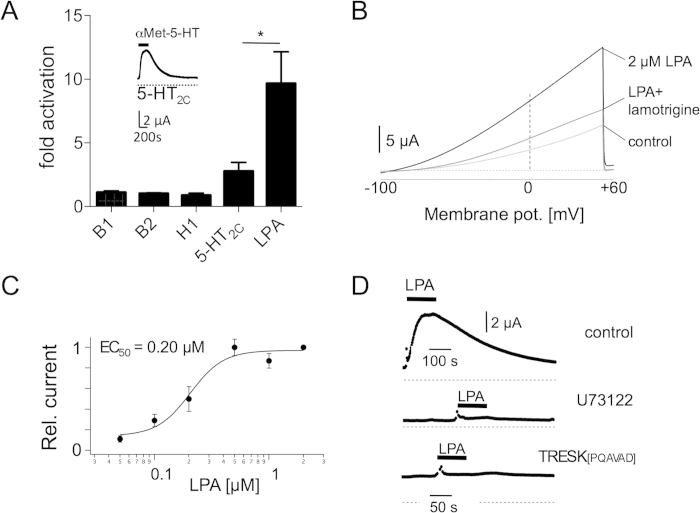
Regulation of TRESK channels by Gq-coupled LPA receptors heterologously expressed in *Xenopus* oocytes. (**A**) Co-expression of 5-HT_2c_, bradykinin B_1_, bradykinin B_2_, histamine H_1_ and LPA_2_ receptors together with TRESK augmented outward currents upon activation with the respective selective agonist. Inset displays representative trace with co-expressed 5-HT_2c_ receptors. (**B**) Ramp recordings from −100 to +60 mV elicited outwardly rectifying currents amplified after application of LPA and blocked by lamotrigine (100 μM) (**C**) Dose-response curve of TRESK activation by LPA recorded at a holding potential of +30 mV with a half-maximal activating concentration of 0.2 μM. (**D**) LPA-induced augmentation of outward currents (upper panel) was abolished either upon incubation with the phospholipase C blocker U73122 (middle trace) or by a mutant lacking the calcineurin binding motif (lower trace).

TRESK current amplitude was increased 2.77 ± 0.69 fold (n = 9) compared to basal current amplitude at a depolarising potential of +30 mV after activation of 5-HT_2C_ receptors with 2.5 nM α-Met-5-HT (Fig. 2A inset). Activation of co-injected B_1_ receptors with bradykinin (100 nM) augmented TRESK currents 1.1 ± 0.12 fold (n = 8) and co-injected B_2_ receptors 1.02 ± 0.06 fold (n = 6), respectively. Histamine (100 nM) augmented TRESK currents less potently 0.88 ± 0.17 fold (n = 12) in comparison to other mediators (Fig. 2A). For each receptor agonist concentrations were chosen to yield maximal activation of TRESK currents.

Phospholipids such as LPA are also secreted during inflammation and were found to modulate the activity of diverse ion channels involved in pain perception^20^,^21^. Co-expression of LPA receptors and TRESK channels augmented basal K^+^ current 9.67 ± 2.48 fold (n = 8, two-tailed t-test, p = 0.046) upon LPA application (0.5 μM) showing this compound to be the most effective activator of TRESK channels (Fig. 2A).

As two homologues of mammalian LPA receptors are endogenously expressed in *Xenopus* oocytes^26^ we tested TRESK activation by endogenous receptors. After sole injection of TRESK cRNA into oocytes, ramp recordings from −100 to +60 mV elicited an outwardly rectifying current that was amplified 6.76 ± 1.42 fold (n = 11) upon application of 0.5 μM LPA. This G-protein coupled receptor activation could be reversed by 100 μM lamotrigine, an inhibitor of TRESK channels^27^ (Fig. 2B). Increasing LPA concentrations augmented TRESK current amplitude with an EC_50_ of 0.2 μM (Fig. 2C). To confirm a Gαq-coupled signalling pathway of LPA-induced augmentation of TRESK currents we used the specific blocker of phospholipase C (PLC) U73122. Pre-incubation of oocytes in a solution containing U73122 significantly reduced LPA-mediated TRESK augmentation to 0.4 ± 0.12 fold (n = 6, two-tailed unpaired t-test, p = 0.006; Fig. 2D, middle trace). In a previous study it has been shown that TRESK can be activated about 10-fold by the calcium/calmodulin-dependent protein phosphatase calcineurin upon binding to the consensus motif PQIVID^28^. To proof whether calcineurin is also necessary for LPA-induced augmentation we mutated this interaction motif by the exchange of two isoleucines with alanine (PQAVAD), as this alteration was shown to inactivate calcineurin binding^28^. Now application of 2 μM LPA totally abolished TRESK current augmentation (n = 12; Fig 2D, lower trace) indicating that this step of signal transduction is essential to regulate TRESK channels by endogenous LPA receptors.

In further experiments we took advantage of endogenously expressed LPA receptors that are necessary and sufficient to regulate TRESK channels.

### Time-locked activation of TRESK and TRPV1 channels

**Figure 3:**
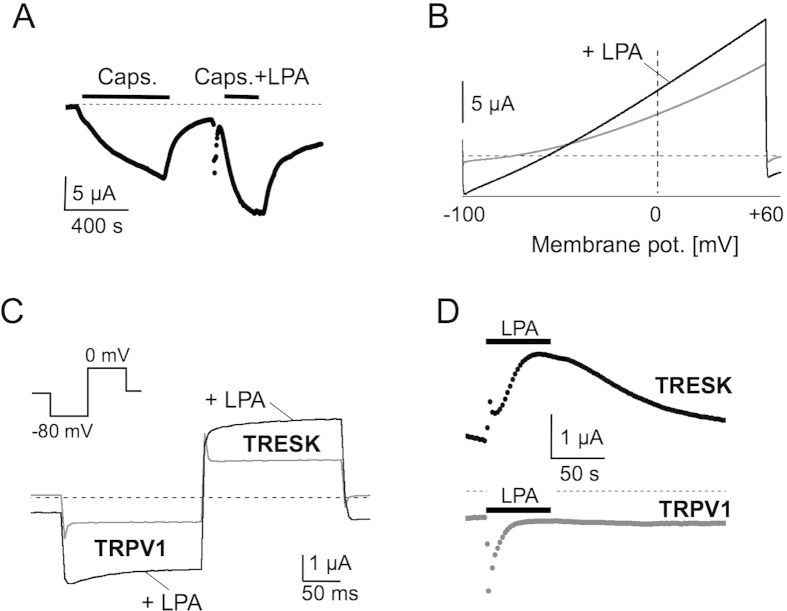
TRPV1 and TRESK are simultaneously activated by LPA. (**A**) LPA augmented capsaicin activated currents in TRPV1 injected oocytes. (**B**) TRPV1 and TRESK co-expressing oocytes displayed an augmented inward and outward current upon application of LPA. (**C**) Pulse protocol to the respective reversal potentials demonstrated time-locked co-activation of TRESK and TRPV1 with LPA expressed in the same oocyte. (**D**) LPA-induced augmentation of TRESK and TRPV1 currents were depicted on a larger time scale. Note the fast deactivation of TRPV1 currents upon sole activation with LPA.

In DRG neurons the capsaicin receptor TRPV1 is one of the major molecular targets to transduce painful stimuli, such as heat, noxious chemicals or acid^22^. Quite recently potent activation of TRPV1 channels by direct interaction with LPA was discovered^21^. Therefore we used the depolarising excitatory TRPV1 current to test the role of TRESK as a regulator of excitability. TRPV1 expression in *Xenopus* oocytes elicited robust inward currents of 6.89 ± 1.64 μA at a holding potential of −80 mV upon application of 2.5 μM capsaicin. Upon co-application of 5 μM LPA, inward currents were augmented to 10.03 ± 2.41 μA without any deactivation kinetics (n = 4, paired t-test, p = 0.028; Fig 3A). The reversal potential of ramp recordings was −2 mV which is close to the calculated Nernst potential of non-selective cation channels (data not shown).

As TRESK channels and TRPV1 receptors are both activated by LPA we next investigated LPA induced effects in the same oocyte co-injected with TRPV1 and TRESK cRNA. Upon application of LPA ramp recordings from these cells displayed an activation of inward currents as well as outward currents with a shift of the reversal potential from initially −81.4 ± 2.7 mV to −55.6 ± 4.06 mV (n = 5; Fig. 3B). Washout of the agonist completely reversed the activation of both currents. To measure TRESK und TRPV1 currents separately in the same cell a specific pulse protocol was applied. Alternative stepping to the reversal potential of either TRESK (−80 mV) or TRPV1 channels (0 mV) allows the recording of TRPV1 currents at −80 mV and TRESK currents at 0 mV. At −80 mV application of LPA (5 μM) led to an 83 ± 20% increase of inward current (n = 14, paired t-test, p = 0.002; Fig. 3C and D) and at the same time point, TRESK outward currents were augmented by 69 ± 15% at 0 mV (n = 14, paired t-test, p = 0.004; Fig. 3C, D). Noteworthy the activation of TRPV1 by LPA displayed prominent deactivation within several seconds. Thus LPA induced co-augmentation of TRPV1 and TRESK in recombinant systems indicates a limitation of excitatory effects during inflammation when TRESK channels are involved in cellular signalling.

### Regulation of excitability in DRG neurons by TRESK channels

#### F-11 cell line

**Figure 4:**
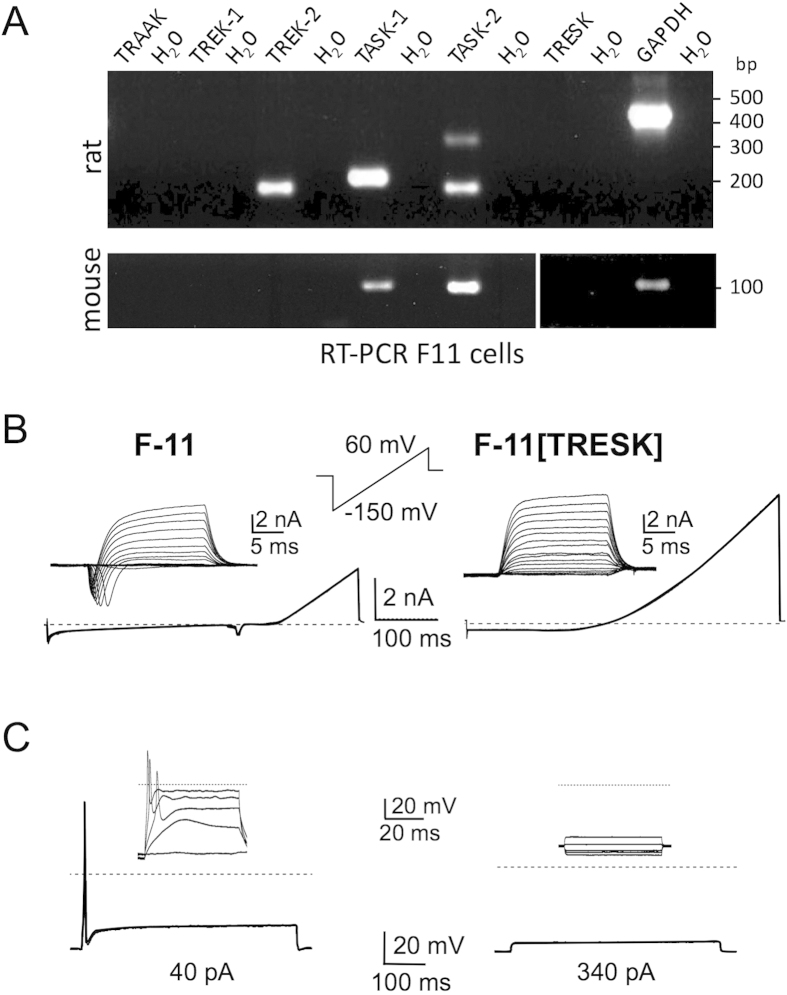
Transfection of F-11 cells with TRESK reduces cellular excitability. (**A**) Expression profile of K2P channels in F-11 cells. PCR-primers specific for transcripts of mouse and rat (as indicated) were used to cover the hybrid genome of F-11 cells. Note that TRESK channels are not expressed. (**B**) Untransfected F-11 cells displayed typical voltage-gated potassium and sodium channels in ramp recordings and in depolarising step recordings (inset, left panel) whereas TRESK transfected F-11 cells displayed a shift of reversal potential to more negative values in ramp recordings and large outward currents upon depolarising steps (inset, right panel). (**C**) Current injection (40 pA) leads to a single action potential in non-transfected F-11 cells (left panel) whereas even high amplitude current injection (340 pA) failed to elicit an action potential in TRESK-transfected cells (right panel). Insets display current steps from −60 to 300 pA.

Next we tried to elucidate how TRESK channels are involved in the regulation of cellular excitability. For this purpose we chose neuronal cells derived from rat DRGs that were hybridised with mouse neuroblastoma cells for immortalisation. These F-11 cells endogenously express voltage-gated HERG K^+^ and Na^+^ channels^29^, Ca^2+^ -activated K^+^ channels^30^ as well as temperature-sensitive TRPV2 channels^31^. However, the expression of TRESK channels in F-11 cells is still unknown. When we conducted a RT-PCR using mouse and rat specific primers with total RNA of F-11 cells we could detect transcripts of TASK-1, TASK-2 and TREK-2, respectively but not TRESK (Fig. 4A). This lack of TRESK channel expression in combination with the ability of firing action potentials renders these cells suitable to investigate the impact of TRESK currents on neuronal excitability.

Ramp-recordings and depolarising pulses evoked inward and outward currents in F-11 cells, which are typical for voltage-gated K^+^ and Na^+^ channels (Fig. 4B). In the current-clamp mode depolarising pulses (40 pA) elicited single action potentials in every recorded cell (Fig. 4C; n = 18). Upon heterologous expression of TRESK in F-11 cells the resting membrane potential showed no significant alteration. However, compared with naive F-11 cells, TRESK-transfected F-11 cells exhibited a larger outward current and a loss of voltage-gated Na^+^ currents. Accordingly, depolarising pulses up to 340 pA failed to elicit an action potential (Fig. 4C; n = 18). Thus, transfection of F-11 cells with TRESK dampens cellular excitability to a large extent even after depolarising these cells with high pulses.

#### DRG neurons

**Figure 5:**
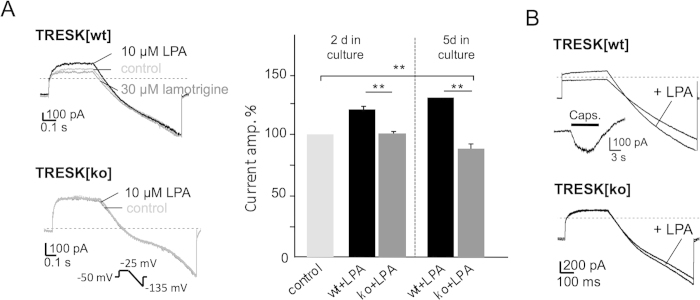
LPA receptors activate IK_SO_ currents in DRG neurons. (**A**) Patch-clamp recordings from TRESK[wt] neurons displayed an increase in current amplitude of IK_SO_ standing-outward currents after application of 10 μM LPA (upper panel, black trace) reversed by lamotrigine (upper panel, grey trace). In neurons from TRESK[ko] mice LPA application had no effect on IK_SO_ (lower panel). Bar graph summarises data from left panel and from neurons 5 days in culture as indicated. (**B**) Recordings from capsaicin positive TRESK[wt] neurons display augmentation of either outward and inward currents upon LPA application, respectively whereas TRESK[ko] neurons display only augmentation of inward currents.

Previous patch-clamp recordings from cultured DRG neurons revealed that about 25% of the standing outward current (IK_SO_) is due to TRESK current^8^. To explore the cooperation of TRESK and depolarising currents such as TRPV1 in native DRG neurons we analysed electrophysiological effects of LPA under voltage-clamp and current-clamp conditions. To avoid clamp artefacts we first recorded currents from neurons that were grown for only 2-3 days, thus having only short outgrowing neurites. IK_SO_ currents of DRG neurons from normal C3H mice (TRESK[wt]) increased from 492.3 to 567.6 nA (19.8 ± 2.9%; n = 11, two-tailed paired t-test, p < 0.0001) after application of 10 μM LPA. This LPA-induced activation was completely reversed by lamotrigine (30 μM; Fig 5A, upper panel). To further corroborate this finding with respect to its specificity for TRESK channels, DRG neurons from functional TRESK[G339R] knockout mice (TRESK[ko])^8^ were cultured and analysed. Under these knockout conditions IK_SO_ was virtually unchanged upon LPA application (437.2 to 442.8 nA; n = 12; Fig. 5A, lower panel). After 5-6 days in culture LPA augmentation of IK_SO_ became even more prominent. Here, the current increased by 28 ± 4.9% (n = 14, two-tailed paired t-test, p = 0.0028) whereas IK_SO_ from TRESK[ko] decreased moderately by 12.95 ± 2.4% (n = 19, two-tailed paired t-test, p = 0.0002; Fig. 5A, right panel) after application of LPA. Most probably the inverse effect in TRESK[ko] DRG neurons resulted from TREK-1 and TASK-1 K2P channels, which also contribute to IK_SO_ in DRG^7^ and were found to be inhibited by LPA^20^.

DRG neurons from TRESK[wt] mice that responded to capsaicin application with depolarising currents showed augmentation of both IK_SO_ and inward (TRPV1) currents when LPA was applied, whereas neurons from TRESK[ko] mice only displayed an augmentation of inward currents (Fig. 5B).

**Figure 6:**
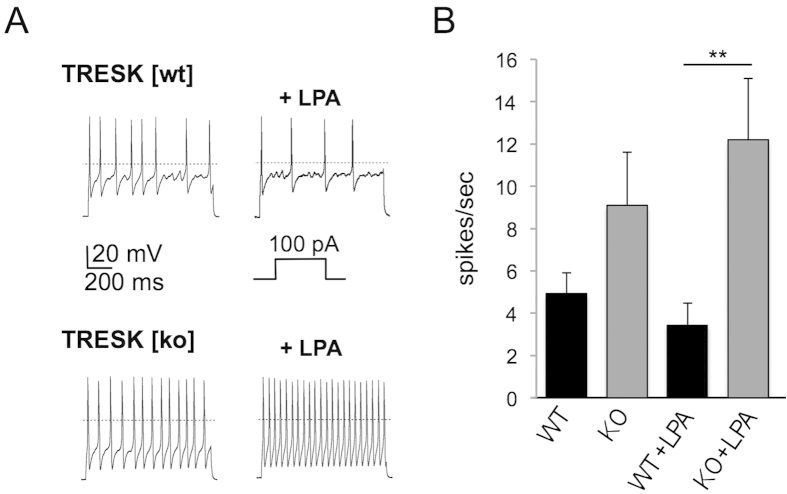
Enhanced excitability of DRG neurons by LPA. (**A**) Current-clamp recordings displayed spike trains from TRESK[wt] and TRESK[ko] neurons upon LPA application. LPA application after depolarising pulses (100 pA) reduced spike frequency in TRESK[wt] neurons (upper trace) and increased spike frequency in TRESK[ko] neurons. (**B**) Bar graph quantifies data shown in (**A**).

To monitor the excitability of DRG neurons from TRESK[wt] and TRESK[ko] mice spike frequencies were compared under current-clamp conditions. To further characterise this effect we paced small diameter neurons from TRESK[wt] and TRESK[ko] mice with depolarising current injections of 100 pA. More precisely, we calculated the current density by monitoring the capacity of each cell we were recording from. TRESK[wt] cells were depolarised with 7 pA/pF and TRESK[ko] cells with 5 pA/pF, respectively, to induce action potential firing. Spike frequency was 4.9 ± 1 spikes/second in TRESK[wt] neurons (n = 14) and 9.1 ± 2.6 spikes/second in TRESK[ko] neurons (n = 10; Fig 6A, left recordings and Fig. 6B). This emphasises the augmented excitability of neurons lacking TRESK currents. Remarkably, under the same conditions LPA application displayed opposing effects. Spike frequencies were attenuated to 3.4 ± 1 spikes/second in TRESK[wt] neurons and augmented to 12.2 ± 2.9 spikes/second in TRESK[ko] neurons (Mann-Whitney U test, p = 0.009; Fig. 6A, right recordings and Fig. 6B). We hypothesise that this effect is due to the activation of TRESK channels in neurons from wildtype animals thereby dampening LPA induced excitability. In neurons from knockout animals LPA only activated depolarising currents (e.g. TRPV1, TRPA1) or slightly reduced hyperpolarising currents (TREK-1 or TASK-1) leading to enhanced excitability.

## Discussion

Pain sensation and the adequate reaction to noxious stimuli are essential for survival. However, the normal physiological function gets lost when pain exaggerates to hyperalgesia during inflammation or chronic pain. Emerging evidence arises that ion channels regulating excitability of afferent nociceptors may constitute an appropriate target for the treatment of pain^32^. Cationic TRPV and voltage-gated sodium channels are the major current components for initial depolarisation after heat stimulation and propagation of action potentials. TRPV1 is predominantly expressed in small-diameter nociceptive neurons from dorsal root ganglia together with other ion channels modulating cellular excitability. Among these, tandem-pore potassium background channels have been identified to be the key players in stabilising the membrane potential and the threshold for action potential firing^1^. As revealed by RT-PCR and means of electrophysiology the K2P channels TREK-2 and TRESK are most abundantly expressed in DRG neurons^7^,^8^. Using immunocytochemistry we could confirm the expression profile with our newly developed, highly specific TRESK antibody. In addition we demonstrate that TRESK appears in neurites and thus is properly targeted to take over sensory function in the periphery. Within the K2P channel family TREK-1, TREK-2 and TRAAK are also significantly expressed in DRG neurons^7^,^8^,^24^,^33^. Both TREK and TRESK channels are regulated by G-protein-coupled receptors^34^,^35^. Upon stimulation of Gαs and Gαq-coupled receptors TREK-1 and TREK-2 are downmodulated by phosphorylation either by Gαs-activated PKA^36^,^37^ or Gαq-activated PKC^38^,^39^. In contrast TRESK channels are activated by Gαq-coupled signalling mediated by elevated intracellular Ca^2+^ and calcineurin-dependent dephosphorylation of the channel. In this former study muscarinic M1 receptors were used to potently deplete intracellular calcium stores followed by augmentation of TRESK currents up to 10-fold when expressed together with the receptor in *Xenopus* oocytes^35^,^28^.

TRESK has been suggested to play a significant role in nociception during inflammation as its transcript levels decreased over time after CFA (complete Freund’s adjuvans)-induced inflammation correlating with spontaneous pain behaviour in rats^24^. On the protein level our experiments with Gαq-coupled receptors identify the LPA receptor as the most effective activator of TRESK currents compared with B_1_, 5HT_2c_ or H_1_ receptors, respectively. A significant increase in TRESK single channel activity was observed in DRG neurons after application of acetylcholine, glutamate and histamine^27^, which is in accordance to our data in recombinant systems.

Apart from well-known mediators that contribute to inflammatory hyperalgesia, oxidised lipids such as LPA have been identified to modulate nociception. During UVB-induced skin inflammation levels of LPA increase and induce peripheral hyperalgesia resistant to treatment with cyclooxygenase inhibitors^40^. TRPV1 channels substantially expressed in pain sensing neurons are activated by direct interaction with LPA^21^. In addition we clearly identify Gαq-coupled LPA receptors to be responsible for TRESK channel activation, because PLC-inhibitors and inhibition of calcineurin interaction with TRESK^35^ completely blocked K^+^ current augmentation by LPA. However, signalling by LPA receptors may also lead to inhibition of K2P channels as TREK-1 currents were inhibited by LPA when recombinantly expressed in *Xenopus* oocytes^20^.

According to the above mentioned channels their level of expression and their mode of regulation determine the excitability of a neuron. In a first step the consequences were demonstrated by co-expression of TRPV1 and TRESK channels in *Xenopus* oocytes. Activation of both channels by LPA is supposed to result in a limited excitatory effect of TRPV1 currents due to hyperpolarising TRESK outward currents. On the other hand an inhibitory effect of LPA on TREK-1 channels would promote the excitation by TRPV1. Considering noxious heat stimuli TRPV1 and TREK-1 are both activated and thus influence excitation to the contrary^22^,^41^, which led to the concept of noxious excitation balanced by hyperpolarising K^+^ currents^42^.

Transfection of DRG-like F-11 cells with TRESK cDNA results in large outwardly rectifying potassium currents that normally stabilise the membrane resting potential. TRESK has a minor impact on the resting potential itself as it is unchanged in non-transfected compared to transfected cells (see also^8^,^43^). Excitability can be altered without any change in resting membrane potential. For example DRG neurons display a higher excitability after sciatic nerve axotomy without significant differences in resting membrane potential^10^. However, TRESK expression inhibits depolarising inputs and depresses the initiation of an action potential. This clearly demonstrates the importance of TRESK channels to cellular excitability. Similarly in cultured trigeminal neurons heterologous expression of TRESK channels significantly reduced the number of evoked action potentials after depolarising pulses^43^. In contrast, transfection of trigeminal neurons with an inactive TRESK mutant showed a dominant negative effect on endogenous TRESK currents and thus increased cellular excitability^12^. In DRG neurons responses to painful mechanical stimuli were significantly increased upon *in vivo* silencing of TRESK channels by intrathecal injection of siRNA^9^. Thus different lines of evidence demonstrate the pain-alleviating effect of TRESK channel activity.

LPA is generated by the action of different enzymes like phospholipase A1 or autotaxin^44^ and is present in a couple of biological fluids like blood plasma and serum as well as in several organs including the nervous system^17^. In DRGs gene expression of LPA receptors has been demonstrated before^45^. In addition to LPA_1_ we could also detect LPA_3_ and exclude the expression of LPA_2_, LPA_4_ and LPA_5_. Although PCR approaches do not differentiate between cell populations co-expression of LPA receptors with TRESK is most likely, as the channel is localised in the majority of DRG neurons^8^. LPA is able to induce peripheral nociception after intraplantar injection into mouse limbs (for review see 17). Direct activation of TRPV1 channels has been considered as a molecular mechanism leading to depolarisation and spontaneous firing of DRG neurons^21^. We could show that simultaneous co-activation of TRESK channels by LPA could down regulate depolarisation-induced spike activity of DRG neurons from normal C3H mice (TRESK[wt]). In contrast, DRG neurons from TRESK[ko] mice lack this compensatory mechanism and depolarisation-induced activity was increased. Analgesic compounds like local anaesthetics normally suppress depolarising currents to avoid the generation of sensory potentials or propagation of action potentials in the periphery. Our findings suggest the alternative possibility to amplify hyperpolarising currents and thereby counterbalance excitation. This complements the concept proposed for TRPV1 and TREK-1 by Eric Honoré^42^ (see above). Whereas TREK-1 inhibition by inflammatory mediators promotes excitation of pain sensing neurons^46^, the activation of TRESK under such conditions reduces the excitatory influence of TRPV1 channels and thus attenuates nociception. The latter effect is assessed to be dominant as TRESK, compared to TREK-1, is the predominant K2P channel present in DRG neurons^8^.

A cognate TRP channel, TRPA1, is also substantially expressed in DRG neurons and may also contribute to this balanced system of opposing currents activated by inflammatory substances^47^. Oocytes injected with TRPA1 displayed a large inward current upon activation by allylisothiocyanate (AITC, 50 μM) that was increased upon co-application of LPA (5 μM) by a factor of 1.35 ± 0.086 (Kollert *et al*., unpublished).

Another line of evidence arises from TRESK mutational studies in familial migraine with aura, which show that a frameshift mutation in the TRESK gene co-segregates with a hereditary form of migraine^11^,^48^. This mutation leads to loss of function with a dominant negative effect on TRESK current amplitude^11^. Less current density of the mutant channel results from improper membrane targeting of the protein, at least in part^11^.

Our study supports the notion that TRESK channels are essentially involved in pain perception in general especially under inflammatory conditions. Recently diverse drug screening approaches identified new compounds that specifically bind and modulate the activity of TRESK channels^49^,^50^,^51^ suggesting TRESK to represent a promising target molecule for the treatment of severe pain disorders such as migraine.

## Methods

### Ethical approval

All experiments were conducted in accordance with the German legislation on protection of animals and were approved by the local animal care committee (Regierung von Unterfranken, Germany).

### Molecular cloning

All experiments with recombinant TRESK channels were performed with the mouse orthologue (GenBank Acc. No. NM_207261) unless indicated otherwise. Plasmid vectors for heterologous expression of TRESK channels in *Xenopus* oocytes were generated as previously described^23^. Accordingly cDNAs of G-protein-coupled receptors LPA_2_ (NM_020028), serotonin 5-HT_2c_ (NM_012765), histamine H_1_ (NM_008285), bradykinin B_1_ (BC_120684), and bradykinin B_2_ (L_26047) were amplified from total RNA of neuronal tissue by RT-PCR and cloned into polyadenylating transcription vector pSGEM. For recombinant protein expression in mammalian cell lines TRESK cDNA was subcloned into pcDNA3 vector. TRESK channel mutant defective of calcineurin binding (TRESK_[PQAVAD]_; 28) was synthesised by QuikChange mutagenesis from Aligent Technologies (Santa Clara, CA) according to the manufacturer’s instructions. Nucleotide sequences of engineered constructs were checked by sequencing service (Eurofin MWG Operon, München, Germany) and ApE analysis software (edited by M. Wayn Davis, UT).

### Gene expression profiling by RT-PCR

Total RNA from DRG of wildtype male C3H mice (8–10 weeks old) and F-11 cell line was extracted with RNeasy mini kit following the manufacturer’s instructions. To remove contaminations of genomic DNA either gDNA eliminator spin columns or RNase-free DNase was applied (Qiagen). The quantity and quality (ratio OD 260/280 > 2.0) of RNA was assessed using NanoDrop UV-spectrophotometer (Peqlab Biotechnologies, Erlangen, Germany). As resolved by agarose gel electrophoresis distinct bands of 28S and 18S ribosomal RNA indicate good quality of the nucleic acid. Only intact RNA samples were used for gene expression analysis.

To verify the expression profile of K2P channels and G-protein-coupled receptors 1 μg total RNA from DRG neurons and F-11 cells was reversely transcribed with iScript (Biorad, CA) in a final volume of 20 μl. Gene specific and intron-spanning primers were applied to selectively amplify cDNA fragments of rat K2P channels (rTRAAK forward 5′-ggagcagcctcatgagcagc-3′, reverse 5′-ggtagtgatgatggtccccg-3′ [231 bp]; rTREK-1 forward 5′-gcagggattatccccttagg-3′, reverse 5′-gatccccaacccagccagtag-3′ [203 bp]; rTREK-2 forward 5′-gcaggggtcagcccggtagg-3′, reverse 5′-gaaaccgaagagcgggatccc-3′ [183 bp]; rTASK1 forward 5′-agctggagcgcgtcgtgctgc-3′, reverse 5′-cccaggctctggaacatgac-3′ [204 bp]; rTASK2 forward 5′-ggatcagtgccctgggcaag-3′, reverse 5′-gttccactcttccgtcaccatg-3′ [182 bp]; rTRESK forward 5′-cagcagctcaagccccagtgg-3′, reverse 5′-cggccaggatgtccccgatgtc-3′ [208 bp]), mouse K2P channels (mTREK-2 forward 5′-tgtggatgtatttttcctacataggtt-3′, reverse 5′-ctcttgggctggcacact-3′[91 bp]; mTASK-1 forward 5′-ctatgccttctacctcct-3, reverse 5′-cccttctgttgtcctggttt-3′ [92 bp]; mTRESK forward 5′-ggggaaggccaggggatgc-3′, reverse 5′-agagcgctcaggaaggaccagt-3′ [303 bp]; mTRESK, QT00168189 [QuantiTect Primer Assay, Qiagen]; mTREK-1, QT00250229; mTRAAK, QT00102445; mTASK-2, QT01047739), mouse LPA receptor isoforms (mLPA_1-1_ forward 5′-ttcgccagaggactatgaggatgt-3′, reverse 5′-tcggccaggaggaggaagaa CT-3′ [208 bp]; mLPA1-2 forward 5′-ttcgccagaggactatgaggatgtct-3′, reverse 5′-tttgtcgcggtaggagtagatga-3′ [253 bp]; mLPA_2_ forward 5′-cagcctgcttgtcttcctactcatg-3′, reverse 5′-gtccagcacaccacaaatg-3′ [177 bp]; mLPA_3_ forward 5′-caacctcctg gccttcttcatca-3′, reverse 5′-gcgcctctcggtattgctgtcctg-3′ [378 bp]; mLPA_4_ forward 5′-tgcggcagcccagagtc-3′; reverse 5′-tcaatgaattttctggaggca-3′ [243 bp]; mLPA_5_ forward 5′-tgcctgtggtagaaaggagc-3′, reverse 5′-tagggaacaacaa ggtcagagc-3′ [287 bp]) and three Gαq-coupled receptors involved in inflammation (histamin H_1_ forward 5′-ggagatccaggcaagggggt-3′, reverse 5′-ccacggtgtgtagcttgcgc-3′ [253 bp]; serotonin 5HT_2c_ forward 5′-cgattgcagccgagtccgtttct-3′, reverse 5′-ccgcagtgcccaggttca-3′ [223 bp]; bradykinin B_2_, forward 5′gcgtccaaatgccctgctcctg-3′; reverse 5′-aaagttattggcgatggtgatgg-3′ [374 bp]).

Using 1 μl cDNA and Taq Polymerase (Qiagen) PCRs were run on a standard thermo cycler (model T3; Biometra, Germany) with the following conditions (total volume 25 μl): initial denaturation step 4 min 94 °C; 30 cycles: 1 min 94 °C, 45 s 58-63 °C, 45 s 72 °C; final elongation step 4 min. 72 °C. Samples were tested for GAPDH expression by PCR (25 cycles) with gene specific primers producing a cDNA fragment of 424 bp (forward 5′-cggcaaattcaacggcacagtcaa-3′, reverse 5′-ctttccagaggggccatccacag-3′) or 100 bp (QT00309099). Fractions of PCR samples were analysed on a 2% agarose gel.

### Cell culture and transfection

With some modifications DRGs were isolated as described previously^52^. Briefly, adult mice anaesthetised with isoflurane were decapitated and DRGs from all levels were dissected. The ganglia were incubated in Dulbecco’s modified Eagle’s medium (DMEM, Invitrogen) containing collagenase Type 2 (10 units/ml) for 120 min at 37 °C with threefold replacement of medium. Individual cells were obtained by treatment with trypsin (10000 U/ml PBS) for 10 min and subsequently triturated with a fire-polished siliconised glass pipette. The cell suspension was centrifuged through a layer of 20% Percoll (Amersham Bioscience, Freiburg, Germany) in PBS to result the neuronal cell fraction in the precipitate. Dispersed cells were plated on poly-L-lysine-coated cover slips and cultured in DMEM supplemented with 10% horse serum and 100 units/ml penicillin-streptomycin (Pen/Strep) at 37 °C with 5% CO_2_. For immunocytochemical analysis DRG neurons from E13.5 mouse embryos were cultured in the presence of 5 ng/ml BDNF, NGF and NT-3 in accordance to the protocol of Jablonka and colleagues with minor modifications^53^.

HEK-293 cells (ATCC CRL-1573) and F-11 cells^54^ were cultured in DMEM supplemented with 10% fetal bovine serum and Pen/Strep at 37 °C with 5% CO_2_. Cells were transfected with polyethylenimine (PEI, Sigma-Aldrich, Germany) at a working concentration of 1 μg/ml. Briefly, cells were plated with a density of 1 × 10^5^ per 35 mm dish and cultured until the following day. For transfection a vigorously stirred mixture of 12 μl PEI, 3 μg plasmid-DNA and 150 μl DMEM w/o serum was incubated for 10 min at room temperature, complemented with 850 μl full culture medium and entirely used to treat pre-cultured cells for 2–3 hours. Supplied with fresh culture medium cells were ready for experiments after 2–3 days.

### Immunocytochemistry

To generate an antibody recognising mouse TRESK channels, a peptide sequence corresponding to amino acids 197–264 (RKQPD…VERSNS) of the mouse TRESK subunit was subcloned into pGEX vector resulting in a fusion construct of the partial intracellular TRESK loop and Glutathion S-transferase (GST). The fusion protein was heterologously expressed in *E. coli* BL21[DE3] and subsequently purified by several steps of column chromatography finished with GST-affinity purification.

Within a period of 18 weeks an adult rabbit was immunised by 4 injections of this antigen (150 μg each). Fourteen days after the final immunisation, a 50 ml serum aliquot was affinity purified on an automated liquid chromatography system using a tandem array of two GST and GST-TRESK affinity cartridges, respectively. The flow-through of the first purification cycle was subjected to five additional purification cycles, each consisting of a loading step and three washing steps followed by elution. The six eluates were pooled and dialysed against PBS. The resulting anti-TRESK antibody was concentrated through ultrafiltration and subsequently sterile filtered. Manufacturing of the antibody was conducted by immunoGlobe GmbH (Himmelstadt, Germany).

For immunocytochemistry HEK-293 cells and DRG neurons were seeded on coverslips and either transfected with mTRESK-eGFP fusion construct (HEK-293) or cultured under normal conditions (DRG). After 2–3 days HEK-293 cells were fixed with 4% paraformaldehyde (PFA) in phosphate buffered saline (PBS) and incubated with blocking buffer (PBS with 10% normal goat serum (NGS) and 0.2% TritonX-100) for 30 min at room temperature. Thereafter coverslips were incubated with rabbit polyclonal TRESK antibody (1:500) in buffer (PBS with 1% NGS and 0.2% TritonX-100) at 4 °C overnight. Following 4 washes with buffer coverslips were incubated with Alexa Fluor 555-conjugated goat anti rabbit antibody (1:1000, Invitrogen) for 2 h and then washed again 4 times with buffer and finally with H_2_O. The coverslips were mounted with Roti-Mount FluorCareDAPI (Roth, Germany) which in addition counterstained nuclei of the cells. Transfected HEK-293 cells were identified by GFP fluorescence. After 1 day in culture embryonic DRG neurons were fixed with 4% PFA, optionally permeabilized with 0.3% TritonX, and washed three times with TBS-T (0.1% Tween20). Prior to primary antibody incubation (rabbit polyclonal TRESK antibody, 1:500; chicken polyclonal neurofilament antibody (heavy chain), 1:5000, Millipore, Badford, MA, AB5539; goat polyclonal TRPV1 antibody, 1:300, Santa Cruz, CA, USA, sc-12498) overnight at 4 °C cells were incubated with 10% BSA (bovine serum albumin) for 1 h at RT. The following day DRG neurons were washed three times with TBS-T (0.1%), each for 5 min, and incubated with appropriate secondary antibodies (Cy3-conjugated donkey anti-rabbit, 1:700; Cy5-conjugated bovine anti-goat, 1:500; DyLight649 donkey anti-chicken, 1:500; purchased from Jackson Immunoresearch, West Grove, PA) for 1 h at RT. Finally, cells were washed three times with TBS-T (0.1%), counterstained with DAPI and embedded in heated (65 °C) mowiol. Imaging was acquired with an Olympus Fluo View FV1000 (HEK-293) or the Leica SP2 (DRG) confocal microscope. Membrane fluorescence of transfected HEK-293 cells was documented by single z-layer pictures whereas images of DRG neurons were presented as average projection stacks with a step size of 1 μm comprising 3 layers. Linear enhancement of images for visual presentation was carried out with MacBiophotonics ImageJ software processing corresponding images consistently.

### Protein preparation and immunoblotting

For protein extraction transfected and non-transfected HEK-293 cells were washed with PBS and harvested by scraping the cell layer and subsequent low speed centrifugation of the cell suspension. Equal amounts of protein extracts (estimated using the BioRad protein assay) were separated on 10% polyacrylamide gels and electroblotted onto Immobilon P membranes (Millipore). Western blots were probed with our new polyclonal rabbit anti-TRESK antibody (1:1000) or monoclonal mouse anti-Actin antibody (1:1000, clone C4, Millipore, Temecula, CA). For detection HRP-conjugated goat anti-rabbit or anti-mouse immunoglobulins (1:10000; Jackson ImmunoResearch Laboratories) were applied and after washing developed with selfmade enhanced chemiluminescence detection reagent (0.1 M Tris pH 8.6, 1.25 mM Luminol, 0.6 mM p-Cumaric acid, 0.01% H_2_O_2_).

### Electrophysiology

For heterologous gene expression in *Xenopus laevis* oocytes, capped run-off poly(A^+^) cRNA transcripts from linearised cDNA of receptors and channels were synthesised and injected into defolliculated oocytes. Cells were incubated at 19 °C in ND96 solution (96 mM NaCl, 2 mM KCl, 1 mM MgCl_2_, 1 mM CaCl_2_, 5 mM HEPES, pH 7.4) supplemented with 100 μg/ml gentamicin and 2.5 mM sodium pyruvate. 48-72 hours after injection two electrode voltage-clamp measurements were performed with a TURBO TEC-10 C amplifier (npi, Tamm, Germany). Stimulation and data acquisition were controlled by Pulse software (HEKA, Gemany). Oocytes were placed in a small volume perfusion chamber with a constant flow of ND96 with 0.1% BSA or ND96 with 0.1% BSA supplemented with different concentrations of LPA. For inhibition with U73122 (Sigma Aldrich) oocytes were incubated in ND96 supplemented with 10 μM U73122 for 20 min before performing voltage-clamp measurements.

In addition, primary cultures from adult DRG neurons and F-11 cells were grown on glass cover slips. Whole-cell recordings^55^ were performed 2–7 days after isolation at room temperature in a bath solution consisting of 135 mM NaCl, 5.4 mM KCl, 1.8 mM CaCl_2_, 1 mM MgCl_2_, 10 mM glucose, 5 mM HEPES, pH 7.4. Patch pipettes were pulled from borosilicate glass capillaries (Kimble Products, UK), and heat-polished to give input resistances of 3–7 MΩ (whole-cell). The pipette recording solution contained 120 mM potassium methansulfonate (CH_3_KO_3_S), 4 mM NaCl, 1 mM MgCl_2_, 0.5 mM CaCl_2_, 10 mM ethylene-bis(oxyethylenenitrilo) tetraacetate (EGTA), 3 mM ATP-Mg, 0.3 mM GTP-TRIS and 10 mM HEPES (pH 7.2). Currents were recorded with an EPC9 (HEKA) patch-clamp amplifier and low pass-filtered at 1-2 kHz. Stimulation and data acquisition were controlled by the PULSE/PULSEFIT software package (HEKA) on a Macintosh computer, and data analysis was performed with IGOR software (WaveMetrics, Lake Oswego, OR).

### Statistics

Data are presented as mean ± SEM (number of cells). Statistical analysis was performed with Prism 6 (GraphPad Software Inc., La Jolla, CA) by using two-tailed paired Student’s t-test after testing for Gaussian distribution with the Kolmogorov-Smirnov test. For non-Gaussian distribution Wilcoxon’s rank test and Mann-Whitney U test were used, respectively. P-values ≤ 0.05 were considered to be significant.

## Additional Information

**How to cite this article**: Kollert, S. *et al*. Activation of TRESK channels by the inflammatory mediator lysophosphatidic acid balances nociceptive signalling. *Sci. Rep*. **5**, 12548; doi: 10.1038/srep12548 (2015).
